# Hyovertebrotomy

**Published:** 1895-03

**Authors:** William Dougherty

**Affiliations:** Baltimore, Md.


### HYOVERTEBROTOMY.

BY WILLIAM DOUGHERTY, V.S.

THIS is an operation rarely performed and one requiring very delicate manipulation. I presume you are all more or less acquainted with the operation as described in text-books.

I never but once performed the classical operation, and then only with partial success, by prolonging the life of the colt for a few days. I wish to report, however, the method employed in a number of cases which I operated upon three years ago and two which I operated upon last year.

My method of operating is quite original and as simple as possible. Three years ago I had three cases, at John's Stables, with the guttural pouches full of pus, all on the near side. The same spring I had five yearlings on a stock farm. The pouches were distended on the near side. One was affected on both sides. One died without being operated upon. I had two other cases the same year. The following year I also had ten cases, and last year two cases. Every case made good recovery. The colts were all trained and trotted in races. The other animals were common workhorses. I heard one gentleman make a remark only a few days ago that the colts all made a little noise when speeded, and he attributed it to the sire, but I presume it must have been due to the thickening of the tissue of the surrounding parts. I never made a mistake or had an accident—save two unimportant ones, and they both occurred last year. One was convalescent, and the last time I attempted to draw off the pus the opening had closed, and in forcing the trocar I must have cut a small artery, as the horse bled from the nose for a few minutes, but no bad results followed. The second case was one in which the tissues were very much thickened, and I did not hit the pouch on the first puncture, but it did no harm.

MODE OF OPERATION. I simply use a trocar and canula. I first make a small incision through the skin with a bistoury. I then puncture with a six-inch trocar and canula, very frequently using the whole length of the canula in large horses. The puncture is made about two inches in front of the bifurcation of the jugular vein and the glosso-facial vein, going through the parotid auricular muscle and the parotid gland into the sac, and allowing the pus to discharge through the canula. I repeat the operation of emptying the sac in twelve or twenty-four hours, as the case may require, and afterward as often as necessary.

It takes, on an average, six or seven days for complete recovery. I have never had any ill affects or sequela following the operation.

BALTIMORE, MD.

